# Costal chondrosarcoma mimicking a breast cancer: Case report and review of literature

**DOI:** 10.1016/j.ijscr.2019.02.009

**Published:** 2019-02-14

**Authors:** Nesrine Tounsi, Ines Zemni, Nawel Abdelwahed, Ichraf Jbir, Lamia Charfi, Radhi Ben Nasser, Riadhe Chargui, Khaled Rahel

**Affiliations:** a Department of Surgical Oncologists, Salah-Azaiez Institute, Boulevard of 9-Avril, 1001 Tunis, Tunisia; b Department of Surgical Oncologists, TAHAR EL MAAMOURI Hospital, Tunisia; c Department of Pathology, Salah-Azaiez Institute, Boulevard of 9-Avril, 1001 Tunis, Tunisia

**Keywords:** Chondrosarcoma, Rib, Case report, Reconstructive surgery

## Abstract

**Introduction:**

Chondrosarcoma (CS) was the third most common primary bone malignancy. Frequently chondrosarcoma was occurring in long bones or in the pelvis. Their localization in the rib is rare.

**Case presentation:**

A 45-year-old male presented to our institution with a lump in the left breast. A physical exam revealed a large, fixed 10 cm mass occupied the entire right breast. Clinical presentation had supposed initially as a sarcoma of the breast. Preoperative unenhanced CT imaging showed well-defined round heterogeneous soft tissue density with hypo dense areas.

A core-needle biopsy was taken, which was suggestive of chondrosarcoma grade II. The whole tumor was excised en-bloc along with 4th, 5th and 6th ribs. Reconstruction of the defect was undertaken with polypropylene plate and ipsilateral pedicle latissimus dorsi muscle flap was placed on the alloplastic mesh. The patient had an uneventful postoperative course and was discharged on the 10th postoperative day. No adjuvant treatment was administrated.

**Conclusion:**

En-bloc resection with appropriate margins and thanks to the evolution of reconstructive surgical techniques used to ensure preservation of respiratory mechanics. All this procedure represents the cornerstone and the only curative option.

## Highlights

• The localization of chondrosarcoma in the rib is rare.
• Prognostic of chondrosarcoma is relatively good.
• Generally, chondrosarcoma is radio- resistant and chemo-resistant.
• The cornerstone and the only curative option for CS was en-bloc resection with appropriate margins.

## 1 Introduction

Malignant primary bone tumors of the thoracic wall are rare [1]. Chondrosarcoma (CS) was the third most common primary bone malignancy after osteosarcomas and multiple myelomas [2]. It arises from cartilage-producing malignant mesenchymal cells [3].

Frequently chondrosarcoma was occurring in long bones or in the pelvis. Their localization in the rib is rare [3,4].

Early diagnosis and surgical treatment with widely negative microscopic margins is considered as the gold standard therapy and offers the best chance for cure.

In this article, we present a patient with thoracic chondrosarcoma centered in the 4; 5 anterior thoracic rib. We also detail our operative technique in our institute and review current literature on this topic.

This work have been made following the SCARE Guidelines [5].

## 2 Case presentation

A 45-year-old male with no known history of cancer presented to our institution with a lump in the left breast that had been gradually increasing for 14 months.

**Fig. 1:**
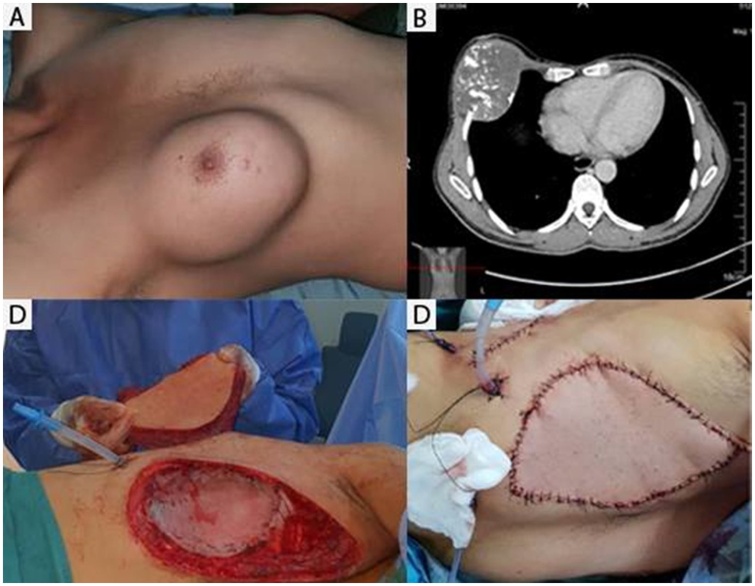
(A) Preoperative image showing the extent of the tumor B) Axial thoracic CT well- showed well-defined mass with peripheral and central calcifications measuring 07 cm × 10 cm × 11 cm. D) Reconstruction of an anterolateral chest wall defect used polypropylene plate and ipsilateral pedicle latissimus dorsi muscle flap.

A physical exam revealed a large, fixed 10 cm mass occupied the entire right breast (Fig. 1-A). The mass itself was adherent to the chest but it did not invade the skin. There was no ulceration, nipple discharge, or retraction. There were no palpable lymph nodes. Clinical presentation had supposed initially as a sarcoma of the breast.

Preoperative unenhanced CT imaging showed well-defined round heterogeneous soft tissue density with hypo dense areas (Fig. 1-B), necrosis and peripheral and central calcifications measuring 07 cm × 10 cm × 11 cm. It was localized in the 4; 5 anterior thoracic rib. The tumor involves the intercostal muscles but respect the lung. CT scan detected no metastatic tumor.

A core-needle biopsy was taken, which was suggestive of chondrosarcoma grade II.

**Fig. 2:**
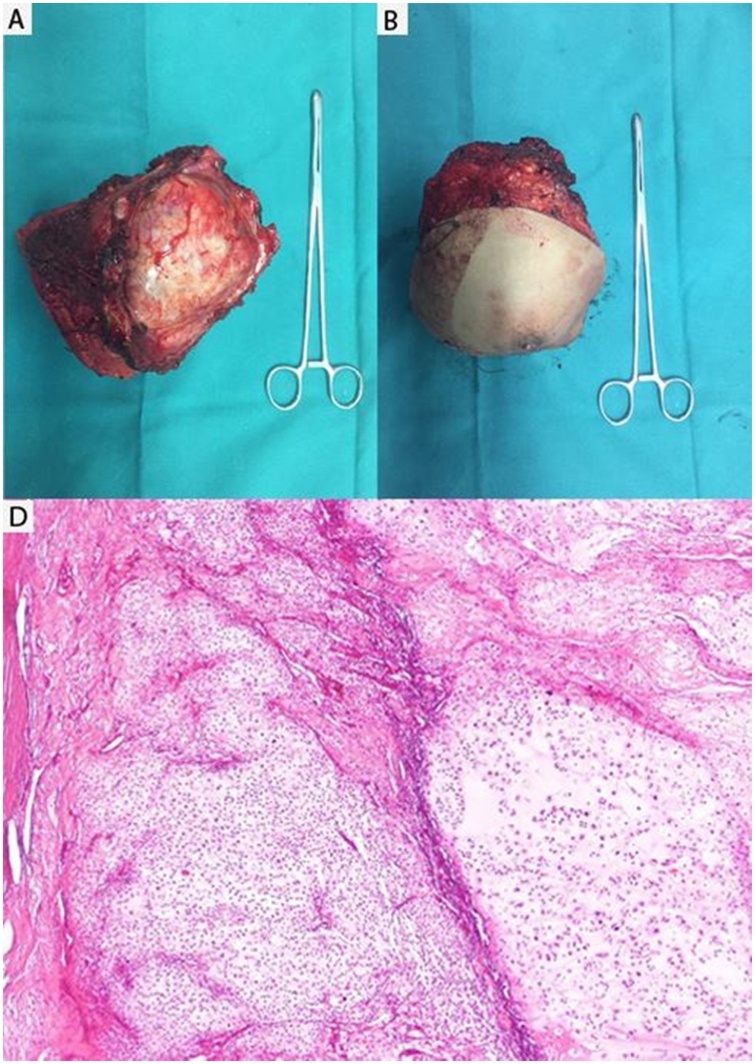
(A-B) front and posterior view of the tumor D) mesenchymal proliferation organized in lobulated architecture with abundant cartilaginous matrix separated by fibrous bands and bony trabeculae bands (HE×100).

Surgery was initiated for wide excision. Intra-operatively, it was found to be arising from the 4; 5 th rib and pushing the lung without invading it. The whole tumor was excised en-bloc along with 4 th, 5th and 6th ribs with a surgical margin of more than 2.0 cm (Fig. 2-A, B). This resection left a defect measuring 23 × 15 cm on the anterior chest wall. Reconstruction of the defect was undertaken with polypropylene plate and ipsilateral pedicle latissimus dorsi muscle flap was placed on the alloplastic mesh (Fig. 1-D). Intercostal drain inserted. The patient was extubated one days after surgery and discharged in 10 days without complication.

In final histopathology report, grossly the tumor has blue-grey color and was attached to bone on one margin and covered by the breast in on one surface. The mass measured 18 cm × 14 cm×13 cm.

Histological section showed mesenchymal proliferation organized in lobulated architecture with abundant cartilaginous matrix and myxoide areas separated by fibrous bands and bony trabeculae bands. Chondrocytes contain enlarged, hyperchromatic nuclei showing high atypia. Mitosis are rare. The margins are free of tumor cells (Fig. 2-D).

The patient had an uneventful postoperative course and was discharged on the 10th postoperative day. No adjuvant treatment was administrated. The patient has now been followed up for 8 months after the excision with no evidence of tumor recurrence.

## 3 Discussions

Chondrosarcoma of the rib is a uncommon malignant tumor [4]. Chest wall chondrosarcomas can occur in the ribs, sternum or both. Chondrosarcoma is frequently observed in men and in the age group 30–60 years [6]. This was consistent with our study.

It is important to keep in mind that a computed tomography (CT) scan is the best imaging of choice.

Recommended to delineate intraosseous or extra osseous involvement and defining vascular or neural involvement [4,7,8].

Surgery considered as the standard treatment with en-bloc resection and a sufficient margin (4 cm margin of normal tissue on all sides). Because chondrosarcoma is not responsive to chemotherapy and radiotherapy [4].

However, in most cases complete resection of large tumor results in a wide defect, requiring most often reconstructive surgery. In rare cases, chondrosarcoma could invaded adjacent organs necessitating a major surgery. such a case was reported by Abraham et al [6], pneumonectomy with thoracoplasty was done due to the extensive involvement of the chest wall and the lung parenchyma. In our present case, tumor did not invaded noble organ. However, surgery left a large defect and this necessitated a reconstructive surgery. Chest wall reconstruction should include stabilization of the bony thorax and coverage of any soft tissue defect. In order to provide rigidity, reconstruction defect should be done with a double sheet of polypropylene mesh with a thin layer of methyl methacrylate paste spread between. Soft tissue reconstruction covering the synthetic materials and skin defect is best accomplished by Muscle and musculocutaneous flap [8].

Prognostic of chondrosarcoma is relatively good with a reported 5-year survival rate ranging between 64 and 80% [9,10].

Most of study concluded that survival was influenced by: tumor diameter <5 cm, tumor location, high histological grade and quality of margins [11,12].

A largest studies of chest wall chondrosarcomas included 96 patients was reported by McAfee et al [11]. In 10 years, recurrence had developed in 50% of patients who had local excision, compared to 17% of patients who had wide resection.

The risk for developing local recurrence or metastasis formation depend in anatomical locations. It was higher, when tumor were localized in sternal, scapular, clavicular, or vertebral [12].

Widhe et al. of the Scandinavian Sarcoma Group studied 106 consecutive patients with chest wall chondrosarcomas. The authors observed that prognostic factors for local recurrence included the surgical margin and histological grade, while prognostic factors for metastases included the histologic grade, tumor size, and local recurrence [9].

Fortunately, in our case there was no involvement of lung parenchyma so a wide resection of all thoracic diseases with appropriate margins it is the rule.

There is no indication of neoadjuvant radiation or chemotherapy in a suitable surgical candidate with a resectable thoracic chondrosarcoma [8].

Generally, chondrosarcoma is radio- resistant and chemo-resistant [1,4]. However, some authors report that chemotherapy improved the survival rate in patients with dedifferentiated chondrosarcomas [13,14]. In addition, Andreou et al [15] reported that patients with metastatic chondrosarcoma who received treatment with radiotherapy (RT) had longer survival than those who received best supportive care. The recent study of Imai et al [16], considers that Carbon ion radiotherapy treatment was a suitable option for unresectable chondrosarcoma.

## 4 Conclusion

The cornerstone and the only curative option for CS was en-bloc resection with appropriate margins and thanks to the evolution of reconstructive surgical techniques, we could ensure the preservation of respiratory mechanics.

## Conflicts of interest

We confirm that we have no conflict of interest.

No financial and personal relationships with other people or organisations that could inappropriately influence (bias) their work.

## Sources of funding

We confirm that we has been no significant financial support for this work that could have influenced its outcome.

## Ethical approval

Ethical approval for this study was obtained from the medical and ethic committee of the Salah Azaiez Institute.

## Consent

Written informed consent was obtained from the patient for publication of this case report and any accompanying images. A copy of the written consent is available for review by the Editor-in-Chief of this journal. Written informed consent was obtained from the patient for publication of this case report and accompanying images.

## Author contribution

IZ, RBN and NT performed the clinical evaluation of the patient. IZ and NT conceived of the report. IZ, LC and IJ performed the literature search and drafted the report. RC and KR critically reviewed and edited the manuscript. All authors read and approved the final manuscript.

## Registration of research studies

N/A.

## Guarantor

The Guarantor: Tounsi Nesrine.

## Provenance and peer review

Not commissioned, externally peer-reviewed.
